# Clinical observation of mild hypothermia combined with intravenous thrombolysis in treating patients with acute cerebral infarction

**DOI:** 10.12669/pjms.37.7.4499

**Published:** 2021

**Authors:** Shaojie Zhang, Lilin Gao, Xuewen Wo, Zhonggong Wang

**Affiliations:** 1 Shaojie Zhang, Department of Neurology, Binzhou People’s Hospital, Shandong 256610, China; 2 Lilin Gao, Department of Neurology, Binzhou People’s Hospital, Shandong 256610, China; 3 Xuewen Wo, Department of Neurology, Binzhou People’s Hospital, Shandong 256610, China; 4 Zhonggong Wang, Department of Neurology, Binzhou People’s Hospital, Shandong 256610, China

**Keywords:** Acute cerebral infarction, Mild hypothermia, Intravenous thrombolysis

## Abstract

**Objectives:**

To investigate the clinical effect of mild hypothermia combined with intravenous thrombolysis in the treatment of acute cerebral infarction.

**Methods:**

Eighty-eight patients with acute cerebral infarction in Binzhou People’s Hospital between May 2018 and August 2019 were randomly selected and divided into a control group and an observation group according to the random number table method, with 44 patients in each group. The control group was given intravenous thrombolysis; the observation group was treated with mild hypothermia (30-35°C) in addition to intravenous thrombolytic thrombolysis. The clinical efficacy, incidence of complications, oxidative stress indexes, inflammatory factor level, neurological function, and mental state of the two groups before and after treatment were compared.

**Results:**

The clinical efficacy of the observation group was significantly better than that of the control group, and the difference was statistically significant (P<0.05). There was no significant difference in the levels of oxidative stress indexes and inflammatory factors between the two groups before treatment (P<0.05). After treatment, the levels of oxidative stress indexes and inflammatory factors of the two groups significantly improved, and the improvement of the observation group was better than that of the control group; the differences were statistically significant (P<0.05). There was no significant difference in the neurological function and mental state between the two groups before treatment (P<0.05). After treatment, the neurological function and mental state of the two groups significantly improved, and the improvement of the observation group was better than that of the control group; the differences were statistically significant (P<0.05). There was no significant difference in the incidence of complications and mortality between the two groups (P>0.05).

**Conclusion:**

Thrombolytic therapy combined with mild hypothermia has a good efficacy in the treatment of acute cerebral infarction. The therapy can improve the neurological function of patients with acute cerebral infarction by significantly improving the oxidative stress index and relieving the inflammatory reaction. Its efficacy is better than single thrombolytic therapy.

## INTRODUCTION

Acute cerebral infarction is a clinical syndrome of neurologic impairment caused by cerebral ischemia and hypoxia, with a high mortality and disability rate; therefore, it is a common emergency and severe disease.^1^,^2^ At present, intravenous thrombolytic therapy within the time window has been written into the diagnosis and treatment guidelines as class I recommendation by many countries, which is one of the most effective methods for the treatment of acute cerebral infarction.^3^,^4^ However, with the extensive development of clinical treatment, it has been found that patients with acute cerebral infarction are prone to a series of adverse reactions and complications after receiving intravenous thrombolytic therapy.^5^ Some researchers have proposed that intravenous thrombolytic therapy can be combined with mild hypothermia treatment, i.e., drug combined with physical cooling treatment in the acute phase, which can protect the brain nerve function of patients and improve the clinical treatment effect.^6^ In addition, research results in China and abroad have confirmed that mild hypothermia treatment has a protective effect on brain cells, and mild hypothermia treatment is conducive to the recovery of neurological function and the improvement of prognosis for patients with acute cerebral infarction.^7^,^8^ However, there are few studies concerning the effect and safety of the combined therapy. Therefore, this study observed the clinical efficacy of intravenous thrombolysis combined with mild hypothermia in the treatment of patients with acute cerebral infarction and its effect on oxidative stress reaction, thus to provide more clinical treatment references for patients with acute cerebral infarction.

## METHODS

Eighty-eight patients with acute cerebral infarction who were received Binzhou People’s Hospital between May 2018 and August 2019 were randomly selected and divided into two groups according to the random number table method, with 44 patients in each group. In the control group, there were 27 males and 17 females; they aged from 45-78 years, with an average age of (62.42±12.07) years; the time from onset to thrombolysis was 1-4.5 h, with an average time of (2.52±0.63) h. In the observation group, there were 28 males and 16 females; they aged 45-79 years, with an average age of (62.76±11.81) years; the time from onset to thrombolysis was 1-4 hour with an average time of (2.47±0.61) hour. There was no significant difference in general data between the two groups (P>0.05); hence, the results could be compared. This study was approved (Ref: 220, Dated: October 10, 2020) by the medical ethics committee of our hospital, and the patients or their families agreed the study and signed informed consent.

### Inclusion criteria

Those meeting the diagnostic criteria of acute cerebral infarction in Chinese guidelines for diagnosis and treatment of acute ischemic stroke 2018 and confirmed by brain computed tomography (CT) or magnetic resonance imaging (MRI) examination;^9^ first onset and the time from onset to thrombolysis ≤ 4.5 hour; no contraindications for intravenous thrombolysis; patients’ family members were informed and signed the consent form; conforming to Declaration of Helsinki.

### Exclusion criteria

With other intracranial lesions, hypotension, uncorrected shock, or cerebral hemorrhage; with coagulation disorder, organ dysfunction, or advanced malignant tumor; with severe combined injury or systemic failure; having a recent history of major surgery or cerebrovascular accident.

The control group was given recombinant tissue plasminogen activator (rt-PA) (Boehringer Ingelheim, registration number S20160054) at a dosage of 0.9 mg/kg. 15% was intravenously injected in the first 30 s, and the remaining drug was pumped in one hour; CT reexamination was performed 24 hour after thrombolysis. After cerebral hemorrhage is excluded, the patient orally took aspirin enteric-coated tablets (Bayer S. p.A., State Food and Drug Administration approval number H20160684; specification: 100 g) at a dosage of 100 mg, once a day. The treatment lasted for two weeks.

In addition to the treatment for the control group, the patients in the observation group were also treated by a medical mild hypothermia treatment instrument (HGT-200, Zhuhai Hejia Medical Equipment Factory). After the patients wore ice caps on their heads, the therapeutic apparatus was turned on. The temperature control tube was inserted to the subclavian vein to control the temperature. The temperature of water was controlled between 6 °C and 12 °C, and the temperature of the tympanic membrane was controlled between 33 °C and 35 °C (measured by an ear thermometer), for 24 h. The far infrared radiation was used for rewarming at the end of the mild hypothermia therapy. The rewarming speed was maintained at 0.5 °C/h. After 12 h-20 h, the body temperature was recovered to 36.5-37.5°C. The vital signs and electrolyte condition were closely monitored in the process of treatment.

The temperature of the cooling instrument was adjusted to keep the water temperature at 6-12°C, and the temperature of the tympanic membrane was kept at 33-35 °C, for three days. The heart rate, blood pressure, and temperature were detected. In case of electrolyte disturbance, coagulation dysfunction, etc., mild hypothermia therapy was terminated. The patient was rewarmed every 1-2 days. The temperature rose slowly, not exceeding 0.1 °C per hour. When the tympanic membrane temperature was 36.5~37.0 °C, the rewarming was stopped.

### Observation index

(1) Neurological function and mental state score: according to the National Institutes of Health Stroke Scale (NIHSS) score,^10^ the total score as 0-42 points, in which NIHSS score ≤ 4 points was defined as minor stroke/mild stroke, and NIHSS score ≥ 21 points was defined as severe stroke; mini-mental state examination (MMSE): 27-30 points was defined as normal, and < 27 points as cognitive dysfunction (21-26 points as cognitive impairment, 10-20 points as moderate cognitive impairment, and 0-9 as severe cognitive impairment. The higher the score was, the better the efficacy was.

(2) Detection of oxidative stress index and inflammatory factors: superoxide dismutase (SOD) was detected with a kit (Shanxi Kanglisheng Pharmaceutical Co., Ltd., China) using the xanthine oxidase method. Malondialdehyde (MDA) was detected with a kit (Chongqing Tonghe Pharmaceutical Co., Ltd., China) using the thiobarbituric acid method. Tumor necrosis factor (TNF) - α) and interleukin (IL)-1β were detected with kits (Chongqing Geruilin Pharmaceutical Co., Ltd., China; Hubei Xinghua Pharmaceutical Co., Ltd., China) by the enzyme linked immunosorbent assay. The above operations strictly followed the instruction books.

(3) Evaluation of clinical efficacy: The clinical efficacy was evaluated according to the NIHSS score.^11^ The decrease of NIHSS score ≥ 91% indicated that the treatment was nearly cured; the decrease of NIHSS score by 46%-90% indicated that the treatment had made a significant progress; the reduction of NIHSS score by 18%-45% indicated that the treatment was effective; and the decrease of NIHSS score < 17% indicated that the treatment was ineffective. The effective rate (%)=(number of nearly cured cases+number of significantly progressive cases+number of effective cases)/total number of cases×100%.

(4) Incidence of complications

### Statistical analysis

The data were processed by SPSS 22.0 statistical software. The measurement data was represented by Mean±SD; the comparison between groups was performed by the t-test. The count data was expressed by percentage; the comparison between groups was performed by the Chi-square test. The difference was considered statistically significant if the value of P was smaller than 0.05.

## RESULTS

The clinical total effective rates of the observation and control groups were 97.56% and 80.49%, respectively. The difference between the two groups was statistically significant (X^2^=4.583, P<0.05, Table-I).

**Table I:** Curative efficacy between the two groups (%).

*Group*	*Observation group*	*Control group*	*X^2^*	*P*
Nearly cured	17(38.6)	10(22.7)	-	-
Significantly effective	18(10.9)	19(13.2)	-	-
Effective	8(18.2)	7(15.9)	-	-
Ineffective	1(2.3)	8(18.2)	-	-
Total effective rate	43(97.7)	36(81.8)	4.583	<0.05

There was no significant difference in the levels of inflammatory factors between the two groups before treatment (P>0.05). After treatment, the levels of inflammatory factors in the two groups significantly improved, and the improvement of the observation group was significantly better than that of the control group. The levels of SOD in the two groups significantly increased, while the levels of MDA, TNF-α, and 1L-1β significantly decreased; the differences were statistically significant (all P<0.05, Table-II).

**Table II:** Levels of oxidative stress indexes and inflammatory factors before and after treatment.

*Group*		*Observation group*	*Control group*	*t*	*P*
SOD (mmol/L)	Before treatment	71.3±12.1	70.8±11.9	0.195	>0.05
	After treatment	93.3±3.9	82.9±5.4	11.637	<0.05
MDA (mmol/L)	Before treatment	10.8±2.1	11.1±2.2	0.776	>0.05
	After treatment	4.5±1.1	7.3±1.4	12.715	<0.05
1L-1β (ng/L)	Before treatment	12.5±2.5	12.7±2.3	0.036	>0.05
	After treatment	5.4±1.2	8.4±1.7	7.443	<0.05
TNF-α(ng/L)	Before treatment	15.6±3.9	15.4±4.1	0.027	>0.05
	After treatment	6.5±0.8	8.3±1.4	12.931	<0.05

There was no significant difference in neurological function and mental state between the two groups before treatment (all P>0.05). After treatment, the neurological function and mental state of the two groups significantly improved; the NIHSS score of the two groups was significantly lower than that before treatment, and the MMSE score was significantly higher than that before treatment. The improvement of the observation group was significantly better than that of the control group, and the differences had statistical significance (all P<0.05, Table-III).

**Table III:** Neurological function and mental state scores before and after treatment.

*Group*		*Observation group*	*Control group*	*t*	*P*
NIHSS score	Before treatment	15.97±3.53	16.01±3.61	0.627	>0.05
	After treatment	5.02±1.25	7.66±1.51	7.134	<0.05
MMSE score	Before treatment	18.23±3.58	18.47±2.79	0.714	>0.05
	After treatment	27.58±2.61	24.51±3.29	4.265	<0.05

There were three cases of transient aggravation, one case of dyspnea, one case of secondary embolism, and two cases of hypotension in the observation group. No patient died in the observation group. The incidence of complications in the observation group was 15.9% (7/44). In the control group, there were three cases of transient exacerbation, three cases of dyspnea, and two cases of secondary embolism. The incidence of complications in the control group was 18.2% (8/44). One patient died in the control group, and the mortality was 0.2% (1/44). There was no significant difference in the incidence of complications and mortality between the two groups (X^2^=0.217, P>0.05; X^2^=1.007, P>0.05).

## DISCUSSION

One of the most evidence-based treatment methods for acute cerebral infarction is intravenous thrombolysis within the time window, which can quickly restore the microcirculation in the infarcted area to alleviate the neurological damage caused by local ischemia.^12^,^13^ In particular, rt-PA has become the first choice of intravenous thrombolytic drugs in the treatment of cerebral infarction in various countries.^14^ However, it has been found that the therapeutic effect of rt-PA intravenous thrombolysis alone is limited, and there were risks of intracranial hemorrhage and ischemia-reperfusion injury in patients during the treatment, which could affect the treatment effect and prognosis.^15^ In recent years, mild hypothermia therapy has made great progress in brain protection and has been gradually recognized by clinical practice.^16^ There are many international experimental studies on the application of mild hypothermia in the treatment of cerebral ischemia, hypoxia, and brain injury. At present, it has been verified that mild hypothermia (28-35 °C) has a definite neuroprotective effect on patients with ischemic cerebral infarction.^17^ An animal experimental study has shown that local mild hypothermia may prolong the thrombolytic treatment time window of cerebral infarction, and its mechanism may be that mild hypothermia inhibits the activation of NF-κB to inhibit neuronal apoptosis, reduce the volume of cerebral infarction, and promote the recovery of neurological function after cerebral ischemia.^18^ A recent clinical study suggests that mild hypothermia treatment can improve the prognosis of patients with acute cerebral infarction, and its mechanism may be that mild hypothermia regulates oxidative stress reaction.^19^

SOD and MDA are common indexes to evaluate oxidative stress reaction. TNF-α and 1L-1β are important inflammatory factors, which can play an important role in inflammatory reaction and aggravate the damage of brain tissue and neurons. The results of this study showed that the post-treatment levels of SOD in the two groups significantly increased after treatment, the levels of MDA, TNF-α, and 1L-1β significantly decreased after treatment, and the observation group had significantly higher levels of SOD and significantly lower levels of MDA, TNF-α and 1L-1β than the control group after treatment, which was consistent with the research results of Yun et al.^20^ A study has shown that mild hypothermia treatment can significantly inhibit the production of oxygen free radicals during cerebral ischemia-reperfusion to reduce the brain reperfusion injury;^21^ in addition, mild hypothermia can down regulate the expression of various inflammatory factors to regulate or inhibit the inflammatory reaction.

In addition, the results of this study showed that the total clinical efficacy of the observation group was significantly higher than that of the control group, and the post-treatment neurological function score of the observation group was significantly lower than that of the control group, which verified that rt-PA intravenous thrombolysis combined with mild hypothermia treatment could significantly improve the neurological deficit of patients, and the findings were consistent with the research results of Hu et al.^22^ The reason for the above result might be that the recanalization after intravenous thrombolysis effectively saved the ischemic penumbra and reduce brain cell necrosis, and mild hypothermia protected brain tissues by reducing brain edema and inflammatory reaction. MMSE score can reflect the mental state of the patients in the later stage. The results of this study showed that the MMSE score of the observation group was significantly higher than that of the control group after treatment, indicating that the combined treatment could significantly improve the mental state of patients.

### Limitations of the study

Although the results of this study showed that rt-PA intravenous thrombolysis combined with mild hypothermia was better than rt-PA intravenous thrombolysis alone in the treatment of acute cerebral infarction, there are still some defects in this study. For example, the duration of mild hypothermia treatment was not studied, and randomized controlled studies with large data sample is needed in the later stage to further verify the authenticity and accuracy of the results.

## CONCLUSION

Intravenous thrombolysis combined with mild hypothermia has a significant effect in the treatment of acute cerebral infarction. The combined therapy can significantly reduce the oxidative stress reaction and inflammatory factor levels and further improve the neurological function and mental state. The treatment has a certain degree of security and is superior to the single thrombolytic therapy within the time window; thus, the therapy is worth clinical promotion.

### Authors’ Contribution:

**SJZ & ZGW:** Study design, data collection and analysis.

**LLG & XWW:** Manuscript preparation, drafting and revising.

**SJZ & ZGW:** Review and final approval of manuscript.

**ZGW:** Is responsible for the accuracy of the study.
